# Development and validation of a recombinant Rap1-based lateral flow immunoassay for rapid serodiagnosis of bovine babesiosis in Kazakhstan

**DOI:** 10.14202/vetworld.2025.1881-1890

**Published:** 2025-07-11

**Authors:** Kanatbek Mukantayev, Zhansaya Adish, Darkhan Kanayev, Laura Tokhtarova, Bisultan Abirbekov, Yergali Abduraimov, Aralbek Rsaliyev, Kanat Tursunov

**Affiliations:** 1 Laboratory of Immunochemistry and Immunobiotechnology, National Center for Biotechnology, 010000, Astana, Kazakhstan; 2 Department of Science and Technology, National Holding Qazbiopharm, 010000, Astana, Kazakhstan

**Keywords:** *Babesia bovis*, bovine babesiosis, enzyme-linked immunosorbent assay, lateral flow immunoassay, rapid diagnostics, recombinant rhoptry-associated protein 1, serodiagnosis

## Abstract

**Background and Aim:**

Bovine babesiosis, caused by *Babesia bovis*, poses significant economic challenges to Kazakhstan’s cattle industry. Early and accurate detection is crucial for interrupting transmission cycles, particularly in regions lacking advanced diagnostic infrastructure. This study aimed to develop a rapid lateral flow immunoassay (LFIA) using a recombinant C-terminal fragment of the recombinant rhoptry-associated protein 1 (rRap1) antigen for the serodiagnosis of bovine babesiosis.

**Materials and Methods:**

A C-terminal fragment (amino acids 345–480) of the *B. bovis* *Rap1* gene was codon optimized and expressed in *Escherichia coli*. The recombinant protein was purified using metal-affinity chromatography and validated through sodium dodecyl sulfate polyacrylamide gel electrophoresis, Western blotting, and nanoflow liquid chromatography-tandem mass spectrometry. A diagnostic evaluation was performed using enzyme-linked immunosorbent assay (ELISA) and LFIA on sera from 102 uninfected and 15 infected cattle, all of which had been pre-tested using polymerase chain reaction. Colloidal gold-protein G conjugates were prepared for LFIA, and test conditions were optimized for antigen concentration and serum dilution. Assay performance was compared with previously published LFIAs.

**Results:**

A 21-kDa rRap1 protein was successfully expressed and demonstrated high specificity to positive control sera. ELISA and LFIA both detected antibodies in 13 of 15 infected samples (sensitivity 86.6%). Specificity was 90.1% for ELISA and 88.2% for LFIA. Receiver operating characteristic analysis showed an area under the curve of 0.83, and Cohen’s Kappa indicated fair-to-moderate agreement between ELISA and LFIA. The LFIA exhibited comparable performance to assays based on merozoite surface antigen 1 or spherical body protein antigens, marking the first successful use of a *B. bovis* Rap1 C-terminal fragment for LFIA-based field diagnostics in Kazakhstan.

**Conclusion:**

The developed rRap1-based LFIA is a promising, field-deployable diagnostic tool for bovine babesiosis, offering rapid results without the need for laboratory equipment. Despite slightly lower sensitivity than ELISA, its simplicity, cost-effectiveness, and specificity support its use in large-scale epidemiological surveillance. Further validation in diverse field conditions and cattle populations is recommended to refine sensitivity and broaden applicability.

## INTRODUCTION

Bovine babesiosis is a globally prevalent tick-borne disease that causes substantial economic losses in the cattle industry [1–3]. Among blood–borne para-sites, *Babesia bovis* induces one of the most sev-ere infections, characterized by the destruction of infected erythrocytes within the microcapillaries of critical organs such as the kidneys, lungs, and brain. The occlusion of these microcapillaries results in multi-organ failure and systemic shock, which frequently culminates in the mortality of economically valuable livestock. Surviving cattle often remain chronic carriers of *B. bovis*, serving as reservoirs that facilitate ongoing transmission of the parasite to naïve animals [4, 5]. Given the absence of effective vaccines and the long-term persistence of asymptomatic carriers, early detection of infected animals is essential to interrupt the transmission cycle [6, 7].

The immunofluorescence antibody test (IFAT) is a standard method for detecting antibodies aga-inst *Babesia* spp. However, its diagnostic utility is limited by cross-reactivity with non-target *Babesia* species, subjective interpretation of results, and low throughput [7, 8]. In contrast, enzyme-linked immunosorbent assay (ELISA) is better suited for large-scale screening and routine diagnostic applications in parasitology [9–11]. A competitive ELISA based on the rhoptry-associated protein 1 (Rap1) antigen has been validated by the International Anti-Epizootic Bureau and, alongside IFAT, represents a widely used diagnostic method. ELISAs are preferred due to their high sensitivity, reproducibility, and scalability in high-throughput diagnostic settings [12]. Despite these advantages, ELISAs are limited by complex multi-step procedures, high costs, and the need for specialized laboratory infrastructure and trained personnel [13].

Lateral flow immunoassays (LFIAs) offer distinct advantages, including ease of use, affordability, independence from laboratory equipment, and rapid results within 10–15 min. Furthermore, these tests exhibit high stability under varying temperature cond-itions. LFIA platforms have been successfully deve-loped for the serological detection of *Babesia bigemina*, *Babesia microti*, and *Babesia gibsoni* using merozoite surface antigen (MSA) and secreted antigen 1 of *Babesia gibsoni* antigens [8, 14–16]. However, the antigenicity of MSA was found to be lower than that of Rap1, largely due to the high genetic diversity among *B. bovis* strains in different regions. Rap1 showed better diagnostic performance in serum samples from Asia compared with those from other parts of the world.

Rap1 is a conserved apical antigen across *Babe-sia* spp., exhibiting notable sequence homology and harboring epitopes recognized by neutralizing antib-odies [17, 18]. However, the full-length Rap1 protein is unsuitable for diagnostic purposes due to its highly conserved N-terminal region, which induces cross-reactivity with antibodies against other *Babesia* spe-cies. In contrast, the C-terminal region exhibits greater sequence variability and reduced cross-reactivity, making it more suitable for serological diagnostics and epidemiological applications. The conserved N-terminal region is typically employed in vaccine development, whereas the C-terminal fragment offers improved diagnostic specificity [19]. While Rap1 has been explored in ELISA formats, its application in LFIA diagnostics for cattle remains understudied, particularly within the Kazakh context.

Although several diagnostic techniques for *B. bovis*, including IFAT and ELISA, have been developed and implemented in various regions, these assays are often limited by infrastructure requirements, complex procedures, and cross-reactivity with other *Babesi*a species. Notably, most LFIA platforms developed for babesiosis rely on MSA or spherical body protein (SBP) antigens, which exhibit reduced antigenicity due to genetic variability among *B. bovis* strains. Despite Rap1’s superior antigenic properties, particularly in Asian strains, and its successful application in ELISA, there is a distinct lack of LFIA systems utilizing the *B. bovis*-specific C-terminal fragment of Rap1, especially in field diagnostics in Kazakhstan. Moreover, the C-terminal region of Rap1 has demonstrated reduced cross-reactivity and increased diagnostic speci-ficity compared with its N-terminal counterpart, yet remains underutilized in LFIA development. To date, no field-deployable LFIA diagnostic tools based on the C-terminal Rap1 fragment have been validated for use in Kazakhstan, despite the region’s high burden of babesiosis and limited access to laboratory diagnostics.

This study aimed to address the diagnostic gap by producing a recombinant C-terminal fragment of the *B. bovis* Rap1 antigen and developing a rapid, specific, and field-applicable LFIA for the serological detection of bovine babesiosis in Kazakhstan. The research focused on codon-optimizing and expressing the Rap1 fragment in *Escherichia coli*, purifying the recombinant protein, and validating its immunochemical properties through sodium dodecyl-sulfate polyacrylamide gel electrophoresis (SDS-PAGE), western blotting, ELISA, and nanoflow liquid chromatography-tandem mass spectrometry (nanoLC-MS/MS). The final goal was to integrate the recombinant antigen into a colloidal gold-based LFIA platform, optimize assay conditions, and evaluate its diagnostic performance using polymerase chain reaction (PCR)-confirmed bovine serum samples. By leveraging the diagnostic potential of the Rap1 C-terminal domain, the study aimed to develop a robust tool for the early detection of babesiosis, adaptable to resource-limited and field settings.

## MATERIALS AND METHODS

### Ethical approval

All animal experiments were conducted after obtaining approval from the Local Ethical Committee of the Research Institute for Biological Safety Problems (No.1, July 14, 2023), in accordance with the Guidelines for the maintenance and care of animals (Interstate Standard, GOST 34088–2017).

### Study period and location

The study was conducted from November 2023 to November 2024 at the National Center for Biotechnology.

### Sample collection

A total of 117 serum samples were collected, including 102 from healthy cows and 15 from infected cows. All animals were tested for the presence of Babesia parasites using the AccuPower *Babesia* PCR kit (#BAB-1115P, Bioneer, Daejeon, Republic of Korea). Samples were obtained from the southern regions of the Republic of Kazakhstan during routine veterinary inspections. Each serum sample was tested twice using ELISA and LFIA.

### Bacterial strains, plasmids, and antibodies

For recombinant Rap1 (rRap1) expression, *E. coli* strains DH5α and BL21 (DE3) (Novagen, Cambridge, UK) were used with pGEM-TEasy (Promega, Madison, WI, USA) and pET28 (Novagen) plasmid vectors. Wes-tern blotting employed monoclonal antibodies against the His-tag conjugated to horseradish peroxidase (HRP) (#34460, Qiagen, Hilden, Germany). In ELISA, anti-bovine HRP conjugate (#A5295, Sigma-Aldrich, St. Louis, MO, USA) served as the secondary antibody. For the LFIA control line, an anti-bovine immunoglobulin G anti-body (#B1645, Sigma-Aldrich) was used.

### Synthesis and cloning of the *Rap1* gene fragment

The amino acid sequence of Rap1 was retrieved from GenBank (accession number AF030062). The gene fragment, codon-optimized for *E. coli* expression, was synthesized using a two-step PCR with Phusion High-Fidelity DNA Polymerase (Thermo Fisher Scientific, Waltham, MA, USA). It was then cloned into the pET28 vector using EcoRI and XhoI restriction sites (Thermo Fisher Scientific).

### Expression and purification of recombinant protein

The recombinant plasmid was transformed into *E. coli* BL21(DE3) cells through electroporation. Trans-formed cells were grown in Lysogeny Broth medium (Sigma-Aldrich, St. Louis, MO, USA) with kanamycin until the optical density (OD_600_) reached 0.5. Protein expression was induced using 0.1 mmol/L isopropyl β-D-thiogalactoside (BioChemica, Darmstadt, Germany) and incubated for 16 h at 23°C. Harvested cells were centrifuged (6,000 × *g*, 4°C, 7 min) and lysed using a UP200S ultrasonic disintegrator (24 kHz, 10 × 10 s pulses, on ice). The recombinant protein was purified using HisTrap nickel-affinity columns (Cytiva, Uppsala, Sweden) and eluted with a linear 20–500 mmol/L imid-azole gradient.

### Characterization of recombinant protein

Protein identity, purity, and integrity were assessed using SDS-PAGE, western blotting, and nanoLC-MS/MS. Protein samples were denatured, loaded on 12% SDS-PAGE gels, boiled (95°C, 5 min), separated at 100 V, stained with Coomassie Brilliant Blue R-250, and destained. For western blotting, proteins were transferred to a 0.45 μm nitrocellulose membrane (GE Healthcare Life Sciences, UK), blocked with 1% bovine serum albumin (BSA) (23°C, 1 h), and washed with phosphate buffered saline with Tween-20 (PBST). His-tag monoclonal antibodies (1:3000 dilution) were added and incubated (23°C, 1 h). The substrate was prepared by mixing 0.01 g of 4-chloronaphthol with 2 mL of methanol, 18 mL of buffer, and 0.01 mL of 3% hydrogen peroxide.

For LC-MS/MS, protein bands were excised, reduced, alkylated, and digested with trypsin (10 ng/μL in 50 mM ammonium bicarbonate). Digests were dried in a SpeedVac, purified using Pierce C18 tips (Thermo Fisher Scientific), separated by high-performance liquid chromatography on an Acclaim PepMap RSLC column (Thermo Fisher Scientific), and analyzed by LC-MS/MS. Spectra were processed using the MASCOT database (Matrix Science Ltd, London, UK).

### ELISA procedure for antibody detection

ELISA was performed using archived sera from PCR-confirmed infected and uninfected cattle. Negative PCR sera were used as controls. The recombinant antigen was adsorbed onto 96-well plates at a concentration of 0.002 mg/mL in 50 mM carbonate-bicarbonate buffer (pH 9.6, 0.1 mL/well) and incubated overnight at 4°C. Plates were washed 4 times with PBST, blocked with 1% BSA (37°C, 40 min), and incubated with serum diluted 1:100 in PBST (0.1 mL/well, 37°C, 1 h). After washing, 0.1 mL of secondary antibody (1:20,000) was added and incubated for another hour. Detection was performed using 3,3′,5,5′-Tetramethylbenzidine (TMB) substrate (Thermo Fisher Scientific) (0.1 mL/well, 23°C, 15 min) and absorbance was read at 450 nm. The ELISA cutoff was set at 3 times the OD of negative controls.

### Synthesis of colloidal gold and protein G conjugation

Colloidal gold nanoparticles (18–20 nm) were synthesized using the Frens method [20]. Briefly, 100 mL of filtered mQ water was boiled, and 1 mL of 1% gold chloride was added. After boiling, 3 mL of 1% sodium citrate was added and maintained for 15 min. The solution was cooled, protected from light, and stored at 4°C.

To determine the optimal protein G concentration, 10 mL of gold suspension (pH 8.5–9.0) was incubated at 23°C for 60 min with protein G, followed by 0.25 mL of 10% BSA and further incubation for 10 min. The susp-ension was centrifuged (8,000× *g*, 30 min), and the pellet was resuspended in phosphate-buffered saline (PBS) with the appropriate BSA concentration.

### Assembly of the LFIA

The colloidal gold-protein G conjugate was dispensed onto PT-R5 membranes (Advanced Microdevices Pvt. Ltd, India) using the AirJet Quanti 3,000 system with an XYZ3050 BioDot dispenser (Bio-Dot, USA). A dilution corresponding to OD520 = 2.0 was used. Test and control lines were applied to CNPC-SS12-L2-H50 nitrocellulose membranes with the FrontLine 1,000 system (BioDot). The test line included purified recombinant protein at concentrations of 0.5–4 mg/mL (in 10% glycerol PBS), while the control line used bovine antibodies (1 mg/mL in 10% glycerol PBS). All solutions were dispensed at 2 μL/10 mm membrane width. Membranes were dried (23°C, 20 h, vacuum thermostat), assembled into lateral flow units, and cut into 3-mm strips under controlled humidity (25%–30%).

### LFIA procedure for antibody detection

LFIA was conducted at ambient temperature (23°C). Serum samples were diluted 1:100 in PBST, and 0.1 mL was placed in a 1.5 mL tube. A test strip was inserted and incubated for 10 min. The results were interpreted using the TSR-100A reader (Hangzhou Allsh-eng Instruments Co., Ltd., China).

### Statistical analysis

All statistical analyses and figure generation were performed using GraphPad Prism version 9.3.1 (GraphPad Software, Boston, MA, USA). p < 0.05 was consid-ered statistically significant. Agreement between ELISA and LFIA results was assessed using Cohen’s Kappa coefficient and receiver operating characteristic (ROC) analysis.

## RESULTS

### Production, purification, and characterization of recombinant proteins

**Figure 1:**
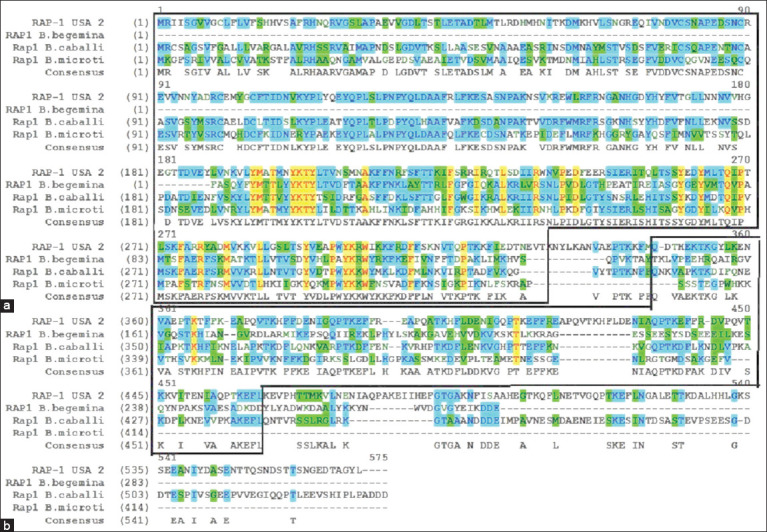
Structural alignment of the amino acid sequences of the rhoptry-associated protein 1 (Rap1) antigen in *Babesia* spp. (a) N-terminal fragment of the Rap1 antigen; (b) selected C-terminal fragment of the rhoptry-associated protein 1 antigen.

A C-terminal fragment of the Rap1 antigen, encompassing amino acid residues 345–480, was selec-ted for LFIA development. The *B. bovis* Rap1 amino acid sequence was aligned and compared with those of *B. bigemina*, *B. caballi*, and *B. microti* (Figure 1). Sequence alignments performed using Vector NTI Advance 11.5.0 (Thermo Fisher Scientific), revealed a high degree of conservation in the N-terminal region of Rap1 across different *Babesia* species. Based on identified differences in the C-terminal region between *B. bovis* and *B. bigemina*, a synthetic gene fragment encoding amino acids 345–480 of *B. bovis* Rap1 was constructed.

**Figure 2:**
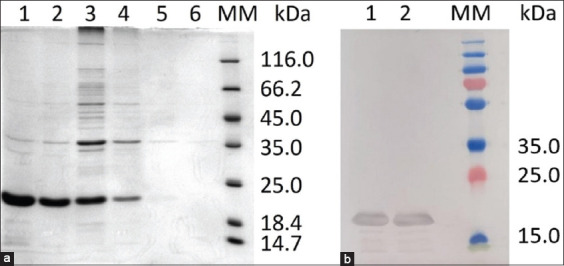
(a) Sodium dodecyl-sulfate polyacrylamide gel electrophoresis and (b) Western blot analyses of recombinant protein expression. Lane 1: 200 mmol/L imidazole; Lane 2: 250 mmol/L imidazole; Lane 3: 300 mmol/L imidazole; Lane 4: 350 mmol/L imidazole; Lane 5: 400 mmol/L imidazole; Lane 6: 500 mmol/L imidazole; MM=Molecular markers.

**Figure 3:**
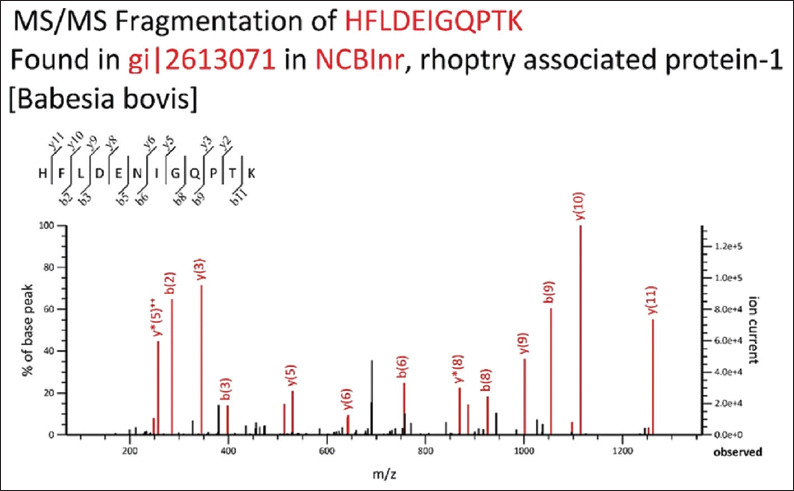
Tandem mass spectrometry of fragmented recombinant protein.

The recombinant antigen was expressed in *E. coli*, and the resulting 21-kDa protein was confirmed through SDS-PAGE and western blotting (Figure 2a). Western blot analysis using anti-His-tagged monoclonal antibodies validated the expression of the expected 21-kDa recombinant protein (Figure 2b). The identity of the recombinant antigen was further confirmed by nanoLC-MS/MS analysis (Figure 3). Mascot peptide analysis of HFLDENIAQPTK yielded a top score of 4,950, uniquely identifying the *B. bovis* Rap1 antigen.

### Specificity determination of recombinant antigens through ELISA

**Figure 4:**
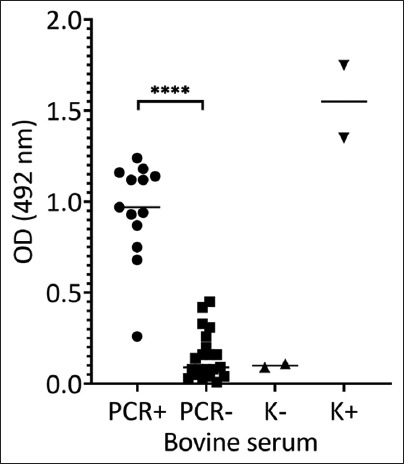
Enzyme-linked immunosorbent assay of recombinant antigen with cattle sera. Polymerase chain reaction (PCR)+ and PCR-: Serum samples from sick and healthy cows diagnosed by PCR. Comparison between groups was performed using a non-parametric t-test.

To evaluate diagnostic specificity, ELISA was conducted on 13 sera from PCR-confirmed infected cattle and 21 from uninfected controls. These same samples were used to determine the threshold level of antibody discrimination between infected and healthy animals. The findings demonstrated that the recombinant antigen is a suitable target for serological detection of bovine babesiosis (Figure 4), achieving 92% sensitivity and 90% specificity.

### Preparation of the colloidal gold-protein G conjugate

Colloidal gold was synthesized and conjugated with protein G to prepare the LFIA detection system. Adsorption efficiency was monitored by observing changes in OD at 580 nm following the addition of 10% sodium chloride. Protein G exhibited peak adsorption at 0.002 mg/mL, with OD values stabilizing from 0.004 mg/mL onward. Based on these observations and the protocol by Sotnikov *et al*. [21], 0.01 mg/mL of protein G was selected for conjugation.

### Development and validation of the LFIA

**Figure 5:**
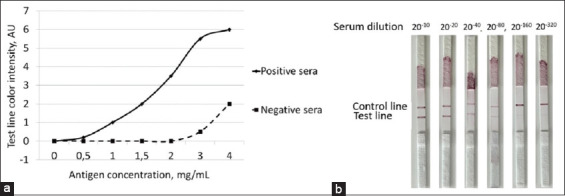
(a) Testing of standard sera using the lateral flow immunoassay based on recombinant rhoptry-associated protein 1 for determining the optimal antigen concentration and (b) when determining the dilution threshold for the serum of sick cattle.

To optimize antigen immobilization on LFIA membranes, various recombinant protein concent-rations (0.5–4 mg/mL) were applied to CNPC-SS12-L2-H50 membranes at 2 μL/10 mm. The colloidal gold–protein G conjugate was applied to PT-R5 membranes at 20 μL/10 mm. Control sera were used at a 1:20 dilution (Figure 5a). Test line signals at 3 and 4 mg/mL antigen were comparable, indicating satu-ration, whereas concentrations of 1 and 1.5 mg/mL yielded weak signals. A 2 mg/mL antigen concentration was identified as optimal, providing a balance of sensitivity and specificity.

The LFIA sensitivity threshold was determined by testing serial dilutions (1:10–1:320) of positive serum (Figure 5b). Detectable signals were observed up to a 1:80 dilution, beyond which signal intensity diminished. These results confirm the LFIA’s capacity to detect antib-odies against *B. bovis* at diagnostically relevant levels.

### Relative sensitivity and specificity of ELISA and LFIA

The performance of ELISA and LFIA was evaluated using 117 serum samples, comprising 102 from uninfected and 15 from infected cattle, all of which were confirmed by PCR. Among the 102 PCR-negative samples, ELISA and LFIA returned negative results for 90 and 88 samples, respectively, yielding relative specificities of 90.1% and 88.2%. Among the 15 PCR-positive samples, both assays detected antibodies in 13 cases, with a relative sensitivity of 86.6% each.

Statistical analysis using a non-parametric t-test showed no significant difference between ELISA and LFIA performance (p < 0.05). ROC analysis yielded an area under the curve (AUC) of 0.83, indicating high diagnostic efficiency. Cohen’s Kappa values ranged from 0.367 to 0.566, suggesting fair to moderate agreement between the two assays (Table 1).

**Table 1:** Comparison of ELISA and LFIA for the detection of specific antibodies against *Babesia bovis* in cattle serum.

Analysis characteristics	Serum samples and PCR	ELISA		LFIA	
	
		Negative	Positive	Negative	Positive
PCR	102	92	10	90	12
Specificity (%)		90.1		88.2	
Kappa value		0.566			
95% CI		0.352–0.781			
PCR+	15	2	13	2	13
Sensitivity (%)		86.6		86.6	
Kappa value		0.367			
95% CI		0.136–0.869			

ELISA=Enzyme-linked immunosorbent assay, LFIA=lateral flow immunoassay, CI=Confidence interval, PCR=Polymerase chain reaction

### Comparative performance with previously developed LFIAs

The diagnostic performance of the developed LFIA was further compared to previously published LFIA platforms (Table 2) [8, 13, 22, 23]. The developed test showed comparable results to LFIA systems based on MSA-1 antigens. Although its overall sensitivity and specificity were slightly lower than those of dual-antigen systems, it is the first LFIA to incorporate the *B. bovis* Rap1 antigen. This represents a novel approach in the field, particularly within the context of Kazakhstan.

**Table 2:** Comparison of the performance of the previously published LFIA and the developed test system.

Authors	Antigens	Specificity (%)	Sensitivity (%)	Advantages and limitations
Lira-Amaya *et al*., [8]	MSA1 *B. bovis*	83	90	Further studies on larger numbers of cattle and in different geographic regions of Mexico are needed.
Stuart Tayebwa *et al*., [13]	Rap1 *B. bigemina*, SPB4 *B. bovis*	99	92	A promising test for farmers and veterinarians in Uganda. Further optimization is required to improve the sensitivity before it becomes readily available for commercial use.
Huang *et al*., [22]	Rap1 *B. caballi*, MSA2 *B. equi*	100	94	A useful field test for simultaneous serodiagnosis of both types of equine babesiosis, although improvements to BceICT and evaluation on a larger scale are needed.
Kim *et al*., [23]	Rap1 *B. bigemina* SPB4 *B. bovis*	91	96	Further studies on larger numbers of cattle and different geographic regions are needed.
LFIA based on the rRap1 antigen	C-terminal fragment of *B. bovis* Rap1	88.2	86.6	Speed and simplicity of diagnosis. Further studies on a larger number of cattle and various climatic and geographical conditions in Kazakhstan are needed.

LFIA=lateral flow immunoassay, rRap1=recombinant rhoptry-associated protein 1, *B. bovis=Babesia bovis, B. bigemina=Babesia bigemina,* *B. cabali=Babesia cabali, B. equi=Babesia equi*, MSA1=Merozoite surface antigen 1

## DISCUSSION

### Diagnostic utility of LFIAs

LFIAs are widely regarded as effective diagnostic tools for protozoan infections due to their simplicity, rapid turnaround time, and cost-effectiveness [8, 24, 25]. Previously, LFIA-based diagnostics for *B. bovis* primarily utilized recombinant SBP-4 and MSA-2c antigens to detect early-stage antibodies [13, 23, 26].

### Antigen design based on Rap1 epitope specificity

The C-terminal region of Rap1 selected in this study contains two tandemly repeated motifs (FREAPQATKHFL) not found in other *Babesia* species. Immunological studies using CD4^+^ T cells have identified two epitopes in the N-terminal and one in the C-terminal region of Rap1, including FREAPQATKHFL. Although this epitope is not considered immunodominant, it is capable of activating CD4^+^ T cells and promoting gamma interferon production [27]. Based on this analysis, a synthetic gene fragment of *B. bovis* Rap1 encoding this epitope was used for the production of antigen.

### Recombinant protein expression and validation

Bacterial expression systems were selected for producing recombinant Rap1 due to their simplicity, scalability, and cost-effectiveness, which make them well-suited for the development of diagnostic antigens for *Babesia* spp. [28–30]. Validation of the expressed recombinant protein through western blotting, nan-oLC-MS/MS, and ELISA confirmed its structural and antigenic integrity. These results are consistent with findings from ELISAs using MSA-1 in *B. bovis* and Rap1 in *B. bigemina* [8, 31, 32]. While the slightly higher sensitivity of ELISA compared to LFIA was observed, this may reflect sample size limitations rather than differences in antigenicity.

### Diagnostic performance and standardization of LFIA

The LFIA was developed using *B. bovis* rRap1, followed by standardized immunochromatographic assay protocols previously used for classical swine fever and gnathostomiasis [33, 34]. Its performance was evaluated using PCR-confirmed sera, yielding a sensitivity of 86.6% and specificity of 88.2%. Although slightly less sensitive than ELISA, these values align with those of existing LFIA systems targeting *Babesia* spp. [8, 31, 32].

### Potential causes of sensitivity limitation and optimization strategies

Reduced LFIA sensitivity may result from residual *E. coli* host proteins co-purified with the recombinant antigen, potentially hindering specific antigen–antibody interactions. This issue could be addressed using imm-unoaffinity purification techniques with Rap1-specific antibodies to increase antigen purity. Furthermore, expression in eukaryotic or yeast systems may enhance protein solubility and immunoreactivity. Another factor that may affect LFIA sensitivity is the interference from non-specific antibodies competing for conjugate binding. The use of quantum dot-labeled protein G could improve signal specificity and reduce nonspecific interactions, thereby enhancing assay performance.

## CONCLUSION

This study successfully developed and validated LFIA based on a recombinant C-terminal fragment of the *B. bovis* rRap1 antigen for the serological detection of bovine babesiosis. The rRap1 antigen, expressed in *E. coli* and confirmed through SDS-PAGE, western blotting, and nanoLC-MS/MS, demonstrated strong immunoreactivity. ELISA and LFIA evaluations using PCR-confirmed cattle sera showed diagnostic sensitivities of 86.6% and specificities of 90.1% (ELISA) and 88.2% (LFIA), respectively. ROC analysis revealed an AUC of 0.83, indicating high diagnostic accuracy.

The developed LFIA offers significant practical advantages: rapid results (within 10–15 min), ease of use, affordability, and field applicability without the need for laboratory infrastructure. These attributes make it particularly suitable for use in remote or reso-urce-limited settings, enabling the timely detection and control of babesiosis outbreaks in endemic regions, such as Kazakhstan.

A key strength of this assay is the use of a species-specific C-terminal Rap1 fragment, which minimizes cross-reactivity with other *Babesia* species. The stand-ardized development process, based on pro-ven lateral flow assay protocols, further enhances reprod-ucibility and scalability.

However, limitations exist. The study involved a relatively small and geographically limited serum panel, which may affect generalizability. Additionally, residual *E. coli* proteins may have contributed to reduced LFIA sensitivity, and background reactivity was observed at higher antigen concentrations. These issues underscore the need for enhanced purification strategies and the consideration of eukaryotic expression systems.

Future research should focus on large-scale validation across diverse cattle populations and regions, refinement of antigen purity using immunoaffinity chromatography, and potential enhancement of signal detection through quantum dot labeling or multiplex formats.

In conclusion, the rRap1-based LFIA represents a promising tool for rapid, field-level diagnosis of *B. bovis* infections. With further optimization and broader validation, this assay can contribute significantly to national surveillance programs and the strategic control of bovine babesiosis in endemic areas.

## AUTHORS’ CONTRIBUTIONS

KM and KT: Designed and planned the study protocol, data analysis and drafted the manuscript. LT: Gene synthesis, cloning, and expression of recombinant antigen. DK: Purification of recombinant antigen and LC-MS/MS spectrometry. BA: Data and sample collection, ELISA, and western blot assays. ZA: Determination of recombinant antigen activity and development of LFIA. YA and AR: Analyzed the data and reviewed and edited the manuscript. All authors have read and approved the final manuscript.
